# Globally accurate potential energy surface for the ground-state HCS(X^2^A′) and its use in reaction dynamics

**DOI:** 10.1038/srep37734

**Published:** 2016-11-29

**Authors:** Yu-Zhi Song, Lu-Lu Zhang, Shou-Bao Gao, Qing-Tian Meng

**Affiliations:** 1 School of Physics and Electronics, Shandong Normal University, Jinan 250014, China

## Abstract

A globally accurate many-body expansion potential energy surface is reported for HCS(X^2^A′) by fitting a wealth of accurate *ab initio* energies calculated at the multireference configuration interaction level using aug-cc-pV*Q*Z and aug-cc-pV5Z basis sets via extrapolation to the complete basis set limit. The topographical features of the present potential energy surface are examined in detail and is in good agreement with the raw *ab initio* results, as well as other theoretical results available in literatures. By utilizing the potential energy surface of HCS(X^2^A′), the dynamic studies of the C(^3^P) + SH(X^2^Π) → H(^2^S) + CS(X^1^∑^+^) reaction has been carried out using quasi-classical trajectory method.

The HCS radical is a fundamental reaction intermediate in combustion processes^1^, which plays an important role in molecular formation processes of interstellar clouds^2^. The transient species HCS and CH_2_S were identified as a reactive intermediate in hydrogen abstraction reaction of methyl mercaptane (CH_2_SH) with fluorine atoms by means of photoionization mass spectroscopy^3^. Kaiser *et al.* demonstrated that the sulfur-containing species are important in Jupiter’s atmosphere^4^,^5^. They carried out experimental and theoretical studies on the reaction C(^3^*P*_*j*_) + H_2_S, which has the following channels,


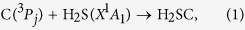



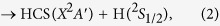



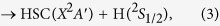



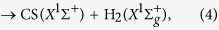



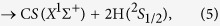


It should be mentioned that Cristina^6^ also studied the isomer pair HCS/HSC and related cations using the coupled cluster (CC) method in conjunction with correlation consistent basis sets ranging in size from quadruple to sextuple zeta for all the species to investigate the near-equilibrium potential energy surface (PES). In 1983, Goddard^7^ firstly predicted the equilibrium geometries of the lowest 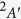 and 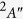 electronic states of HCS using the single-double configuration interaction (SDCI) method with a double-zeta basis. Subsequently, Pope *et al.*^8^ performed a theoretical study of fragmentation and rearrangement processes of H_2_CS and H_2_CS^+^ radicals. Moreover, the HCS/HSC and HCS^+^/HSC^+^ isomerizations were investigated at the CI level in conjunction with triple-zeta basis sets. Stoecklin *et al.*^9^,^10^,^11^ investigated two analytic models of the lowest PES of the reaction SH(*X*^2^Π) + C(^3^*P*) → 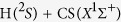 using the MNDO/CI method, three cols connect the HCS to HSC and ground-state products. Potential energy, electric dipole and transition moment functions were calculated by Senekowitsch *et al.*^12^ for the X^2^A′ and A^2^A′′ electronic states of the HCS radical at the multiconfiguration self-consistent field (MCSCF) CI level. For the X^2^A′, the calculated equilibrium geometries are: R_CH_ = 1.083 Å, R_CS_ = 1.573 Å, and *α*_HCS_ = 131.8°, for the A^2^A′′, R_CH_ = 1.063 Å, R_CS_ = 1.557 Å, and *α*_HCS_ = 180°, respectively. They also reported that in linear geometry the two lowest X^2^A′ and A^2^A′′ states become degenerate components of the ^2^Π state and exhibit a strong Renner-Teller coupling effect. HCS, HSC, and the corresponding cationic species were also investigated by Curtiss *et al.*^13^, who carried out a theoretical study of the organo-sulfur systems CSH_n_ (*n* = 0–4) and CS_n_ (*n* = 0–5). The dissociation and ionization energies, and enthalpies of formation were calculated. Ochsenfeld *et al.*^14^ investigated the reaction of atomic carbon with H_2_S using CC theory. In the same year, Chen *et al.*^15^ presented the hyperfine structures of the HCS and isovalent HCO, HSiS and HSiO radicals using the density functional theory (DFT) and multi-reference single and double excitation configuration interaction (MRSDCI) methods. In 2004, Voronin^16^ reported a global analytical PES corresponding to the lowest adiabatic X^2^A′ state of HCS using a grid of 1357 energy points calculated at the MRCI level in conjunction with the aug-cc-pV*T*Z basis set. By employing the PES, the main stationary points of the X^2^A′ surface were also evaluated. Mitrushchenkov *et al.*^17^ performed theoretical studies on HC_n_S (n = 1–12) radicals in the ^2^Π electronic ground state using the Hartree Fock (HF), complete active space self-consistent field (CASSCF) and MRCI methods with different basis sets. Habara *et al.*^18^,^19^,^20^,^21^ detected microwave transition of the HCS, HSC, H^13^CS and HS^13^C radicals in the X^2^A′ ground electronic state.

The major goal of the present work is to obtain an accurate global adiabatic PES for the ground state of HCS based on many-body expansion (MBE) scheme. Both aug-cc-pV*Q*Z (AV*Q*Z) and aug-cc-PV5Z (AV5Z) atomic basis sets have been employed. In order to improve the accuracy of PES, such obtained *ab initio* energies are then extrapolated to the complete basis-set (CBS) limit^22^,^23^,^24^,^25^,^26^,^27^. Based on the adiabatic PES, the reaction dynamics of C + SH reaction were investigated using the quasi-classical trajectory (QCT) method.

## Results

### Features of HCS(X^2^A′) potential energy surface

**Figure 1:**
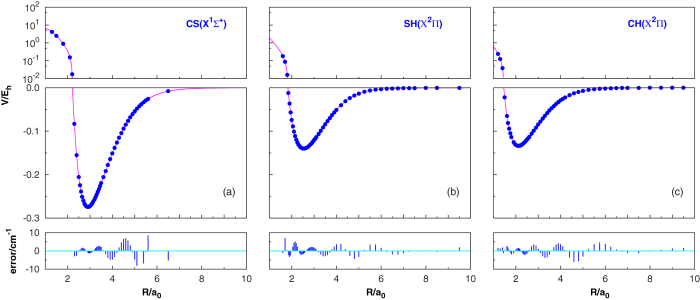
Potential energy curves of CS(*X*^1^∑^+^), SH(*X*^2^Π) and CH(*X*^2^Π), and the differences between the fit and *ab initio* points.

Table 1 shows the characteristics of the CS(*X*^1^Σ^+^), CH(*X*^2^Π) and SH(*X*^2^Π), including equilibrium geometries, dissociation energies, vibrational frequencies, and spectroscopic constants. Other theoretical^22^,^23^,^24^,^25^,^26^,^27^,^28^,^29^,^30^,^31^,^32^ and experimental^33^ values are also gathered in this table for comparison. It can be found that the present values are in good agreement with results from other literatures. The obtained in the present work equilibrium internuclear distances, vibrational constants, and dissociation energies reproduce well the available experimental and theoretical results. Shown in Fig. 1 are the CS(*X*^1^Σ^+^), CH(*X*^2^Π) and SH(*X*^2^Π) potential energy curves (PECs). It can be seen from this figure that the modeled PECs accurately mimic the *ab initio* energies, with the maximum error being smaller than 10 cm^−1^, which shows smooth and accurate behavior both in short and long regions. In order to evaluate the fitting quality of the present PECs, we calculate the root-mean square derivation (rmsd) using the following equation:


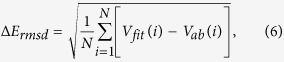


where *V*_*fit*_(*i*) and *V*_*ab*_(*i*) are the *i*-th energies from fitting and from the *ab initio* calculation, respectively; *N* is the number of points employed in the fitting procedure. The values of Δ*E*_*rmsd*_ are 3.89, 2.03, 4.45 cm^−1^ for SH, CH, CS, respectively, which show that the fitted PECs are of a high quality.

**Figure 2:**
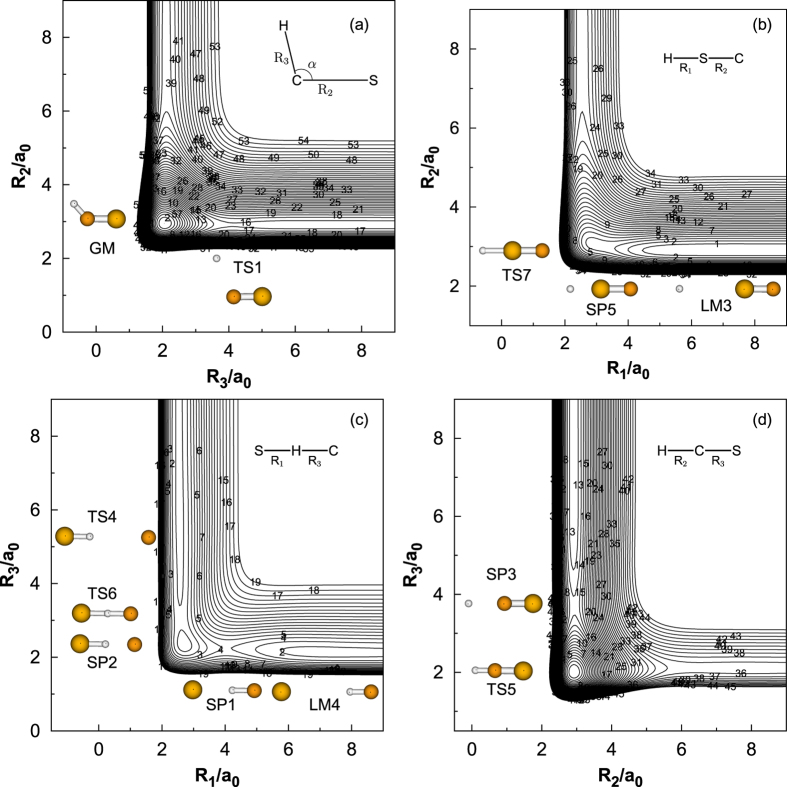
Contour plot for the ground-state PES of the HCS system. Contours are spaced by 0.006 *E*_h_, starting from −0.3614 *E*_h_ in panel (a), −0.2748 *E*_h_ in panel (b), −0.1449 *E*_h_ in panel (c), and −0.3424 *E*_h_ in panel (d).

Figure 2 illustrates the major topographical features of the HCS(X^2^A′) PES. The salient features of these plots are the most relevant stationary points for the title system. Clearly, the variation of the contours is quite smooth in the whole configuration space. Most notable in Fig. 2(a) is the global minimum (GM) with the bending angle being fixed at its equilibrium (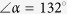), which locates at *R*_1_ = 4.596 *a*_0_, *R*_2_ = 2.953 *a*_0_ and *R*_3_ = 2.062 *a*_0_ (*R*_1_ is the SH interatomic separation, *R*_2_ the CS interatomic separation, while *R*_3_ the CH interatomic separation), and the well depth is −0.3614 *E*_h_ relative to the H(^2^*S*) + S(^3^*P*) + C(^3^*P*) asymptote. In this panel, two valleys are distinguishable: the deeper one at the bottom corresponds to the H(^2^*S*) + CS(X^1^Σ^+^) asymptote, and the valley on the left corresponds to the C(^3^*P*) + SH(*X*^2^Π) asymptote. Observing the Fig. 2(a), one can find that the H(^2^*S*) + CS(*X*^1^Σ^+^) asymptote is bellow the C(^3^*P*) + SH(*X*^2^Π) asymptote, and it is above the global minimum. A closer observation reveals in this figure that there exists no barrier toward the dissociation of HCS(*X*^2^A′) at this configuration into S(^3^*P*) + CH(*X*^2^Π), and there is a transition state (TS1) connecting the global minimum with the H(^2^*S*) + CS(*X*^1^Σ^+^) asymptote. The different collinear configurations are shown in Fig. 2(b–d). In previous works^5^,^8^,^16^,^20^, one local minimum which denoted as LM1 is reported on the PES of HCS(*X*^2^*A*′). However, four local minima (LM1-LM4) are found on the present PES, with two of them being in linear structures. For convenience of discussion, the properties of all stationary points are collected in Table 2, including the internuclear distances, energies and vibrational frequencies. The global minimum for the HCS ground state from our CBS PES is predicted to be located at *R*_1_ = 4.596 *a*_0_, *R*_2_ = 2.953 *a*_0_ and *R*_3_ = 2.062 *a*_0_, differing 0.044 *a*_0_, 0.027 *a*_0_, and 0.008 *a*_0_ from that of ref. 16, which is based on 1357 grid points with AV*T*Z basis set. While the internuclear distances of the *R*_1_, *R*_2_, and *R*_3_ differ from the experimental results^19^ obtained by 0.007 *a*_0_, 0.0003 *a*_0_, and 0.023 *a*_0_, showing a high accuracy. It can be concluded from Table 2 that LM2-LM4 are all in long range, and both LM2 and LM3 correspond to the (H^2^(S) + CS(*X*^1^Σ^+^) asymptote, while LM4 corresponds to the S(^3^*P*) + CH(*X*^2^Π) asymptote. Figure 2(b) shows the contour plot of H-S-C linear stretching, in which the van der Waals local minimum (LM3), second-order saddle point (SP7), and transition state (TS7) can be observed. It is interesting to notice that the energy of the H(^2^*S*) + CS(*X*^1^∑^+^) asymptote is lower than the C(^3^*P*) + SH(*X*^2^Π) asymptote energy level, which indicates that the reaction H(^2^*S*) + CS(*X*^1^∑^+^) → C(^3^*P*) + SH(*X*^2^Π) is an endothermic one. The notable features in panel (c) are another van der Waals local minimum (LM4), two second-order saddle points (SP1 and SP2), and there are two transition states (TS6 and TS4). In the last panel, there is a deep well (TS5) about −0.3486 *E*_h_, located at *R*_1_ = 4.928 *a*_0_, *R*_2_ = 2.916 *a*_0_ and *R*_3_ = 2.021 *a*_0_, which can be seen from Table 2. The TS5 and the CH(*X*^2^Π) + S(^3^*P*) asymptote are connected by a second-order saddle point (SP3).

**Figure 3:**
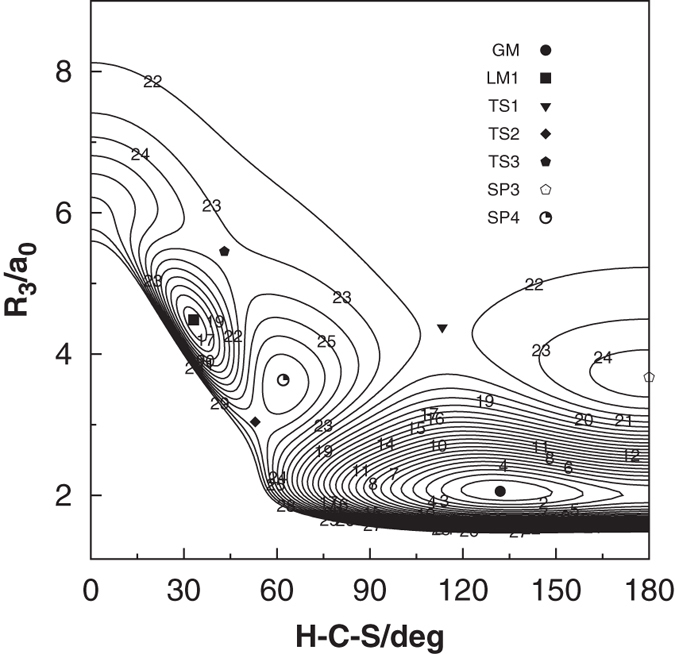
Contour map of the HCS PES plotted as a function of the C-H bond distance and the ∠HCS with an optimized CS bond length (2.9078 a_**0**_). The contours are spaced by 0.004 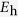, starting from −0.3614 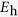.

In order to illustrate the other stationary points on the new PES, a contour plot as a function of the C-H bond distance and the 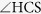 is displayed in Fig. 3, in which the CS bond distance are optimized. The stationary points not appearing in Fig. 2 can be observed in Fig. 3, which are two deep minima (GM and LM1) corresponding to the stable HCS and HSC intermediates and the isomerization transition state (TS2) connecting these two minima is clearly seen from the new PES. The barrier hight of TS2 is calculated to be 21.6 kcal mol^−1^ above the LM1, which is found to be well consistent with the results of Stoecklin *et al.*^9^. The GM well is found to be 37.8 kcal mol^−1^ bellow the LM1 well. The energy compares well with the result reported by Voronin^16^. It is interesting to notice the existence of an entrance barrier for the H addition pathway to the CS molecule (or an exit barrier for the HCS/HSC → H + CS dissociation). In another words, the TS1 connects the global minimum (GM) with the CS(*X*^1^Σ^+^) + H(^2^*S*) asymptote, while TS3 connects the local minimum (LM1) with the CS(*X*^1^Σ^+^) + H(^2^*S*) asymptote. A closer examination shows that the LM1 on the new PES is16.75 kcal mol^−1^ lower than the CS(*X*^1^∑^+^) + H(^2^*S*) asymptote, while Stoecklin *et al.*^10^ reported that the LM2 is 5.1 kcal mol^−1^ above the CS(*X*^1^∑^+^) + H(^2^*S*) asymptote. The difference may be due to the different quality of PES. All major topographical features of the analytical potential energy surface function (APES) are probably better viewed in a relaxed triangular plot^34^ utilizing scaled hyperspherical coordinates (β* = *β*/*Q* and γ* = *γ*/*Q*), where the *Q*, *β* and *γ* are defined as


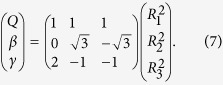


**Figure 4:**
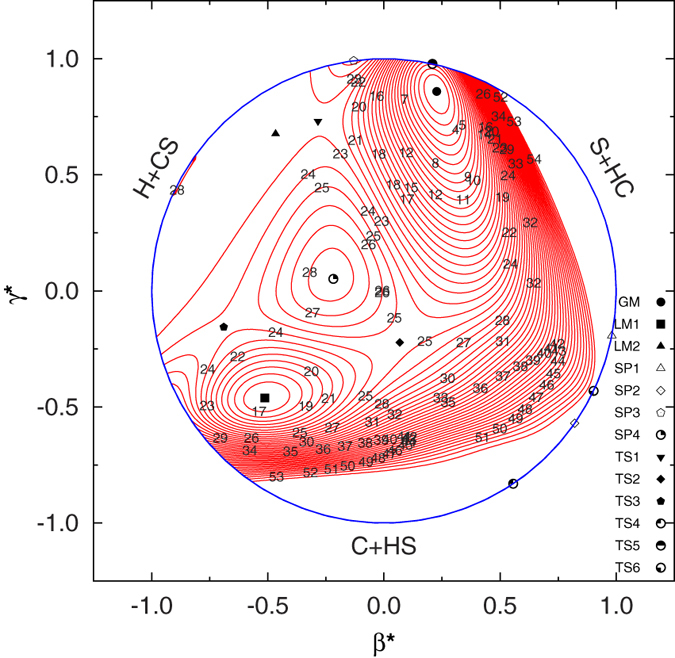
Relaxed triangular plot of new PES in hyperspherical coordinates. The location and symmetry of all stationary points are displayed. Contours equally spaced by 0.004*E*_h_, starting at −0.3613*E*_h_.

As shown in Fig. 4, such a plot depicts in a physical way all the stationary points discussed above.

**Figure 5:**
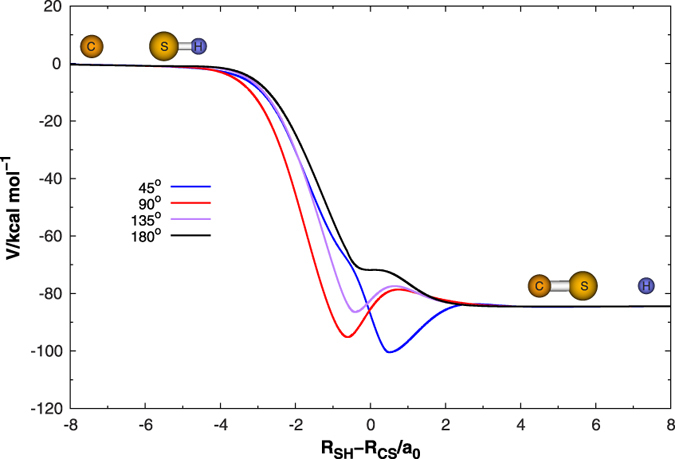
Minimum energy path for the C(^3^*P*) + SH(*X*^2^Π) → H(^2^*S*) + CS(*X*^1^∑^+^) reaction calculated on new PES as a function of R_SH_ − R_CS_ at the 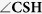 angle 45°, 90°, 135°, and 180°.

The minimum energy paths (MEPs) in different configurations (at a fixed 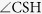 angle) are shown in Fig. 5. These MEPs represent the potential energy of HCS as a function of a suitable reaction coordinate defined as 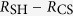, where *R*_SH_ and *R*_CS_ are the SH and CS internuclear distances, respectively. As shown in Fig. 5, *R*_SH_ approaches the SH equilibrium distance when the values of the reaction coordinate are in large negative values, whereas large positive values of the reaction coordinate correspond to *R*_CS_ approaching the CS equilibrium distance. It is shown that the reaction C(^3^*P*) + SH(*X*^2^Π) → H(^2^*S*) + CS(*X*^1^∑^+^) is highly exothermic, with the exothermicity being ∼84.3 kcal mol^−1^, while Stoecklin *et al.*^10^ reported the exoergicity which took into account the zero-point energy is 82.7 kcal mol^−1^. It can be seen from this figure that the well is becoming shallow from 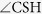 angle 45° to 180°, and the deepest well is ∼16.2 kcal mol^−1^ relative to the H(^2^*S*) + CS(*X*^1^∑^+^) asymptote with the *R*_SH_ = 3.483 *a*_0_ and *R*_CS_ = 2.980 *a*_0_. Note further that the MEPs on the present PES has a shallow potential barrier in H(^2^*S*) + CS(*X*^1^∑^+^) asymptote at 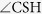 angle 90° and 135°. This barrier can be assigned to be TS3 which connects the LM1 with the CS(*X*^1^∑^+^) + H(^2^*S*) asymptote, with the barrier hight being calculated to be 3.6 kcal mol^−1^ higher than the CS(*X*^1^∑^+^) + H(^2^*S*) asymptote. However, when the angle becomes smaller, the CS(*X*^1^∑^+^) + H(^2^*S*) asymptote and GM are connected by the TS1, which is only 0.25 kcal mol^−1^ higher than the CS(*X*^1^∑^+^) + H(^2^*S*) asymptote.

### Exploratory dynamic studies

**Figure 6:**
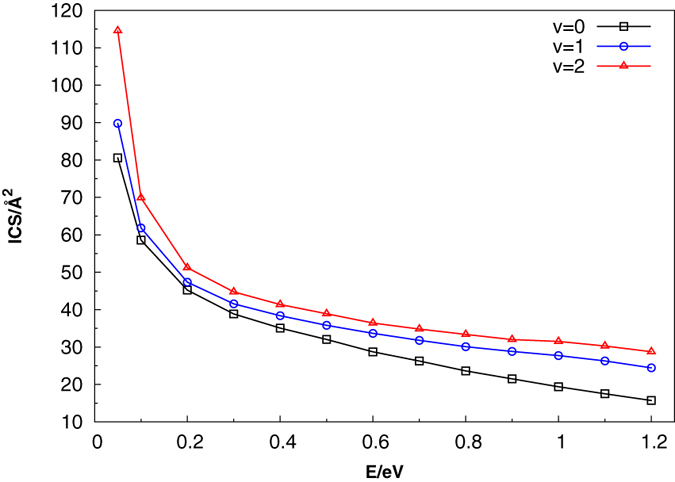
Calculated integral cross sections as a function of collision energy for the C + SH(*v* = 0, 1, 2; *j* = 0).

Based on the present HCS(X^2^A′) PES, we have calculated the integral cross-section (ICS), differential cross-section (DCS) and rate constants of C + SH. Figure 6 presents the QCT ICS as a function of different collision energies (0.05–1.2 eV) for C(^3^*P*) + SH(*X*^2^Π) (*v* = 0, 1, 2; *j* = 0) → H(^2^*S*) + CS(*X*^1^∑^+^) reaction. An obvious decline can be seen in Fig. 6 as the collision energy increases, which is ascribed to the deep well and barrierless aspect of the present HCS(X^2^A′) PES. As the collision energy increases, the ICS curve shows a rapidly decreasing trend from 0.05 eV to 0.2 eV, and relatively slow circumstance between 0.2 eV– 1.2 eV when *v* = 0. The trend of this curve is very similar to other exothermic reactions^35^,^36^,^37^,^38^. In order to further investigate the effect of the reactant SH vibrational excitation on this reaction, we plotted the ICSs of C + SH(*v*, *j* = 0) reaction for *v* = 0, 1, 2 in Fig. 6. We can see that the ICSs for the three vibrational states all decrease rapidly as the collision energy increases. As the vibrational quantum number increases, the ICSs for the *v* = 1, 2 are all higher than that of the ground vibrational state, and the larger the vibrational quantum number, the higher the related ICSs. Thus, it can be concluded that SH vibration enhances the reactivity.

The so obtained ICSs are then used to calculate the rate constant of the title reaction at 300 K, which is 5.12 × 10^−13^ cm^3^ molecule^−1^ s^−1^. Unfortunately, as far as we know, there is no experimental work reported on the rate constant. Thus, a direct comparison cannot be made between them. While, Voronin *et al.*^16^ and Stoeclclin’s *et al.*^11^ calculated the rate constant at the same temperature. The results of Voronin^16^ is 1.745 × 10^−13^ cm^3^ molecule^−1^ s^−1^. The difference may be due to the different characteristics of the present PES and that of Voronin *et al.*^16^.

**Figure 7:**
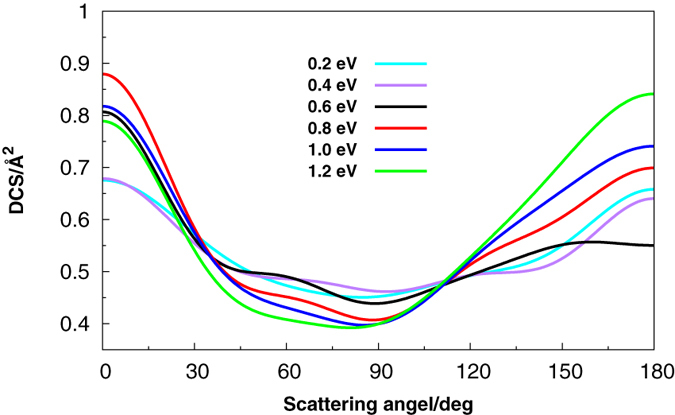
Differential cross section at different collision energies.

**Figure 8:**
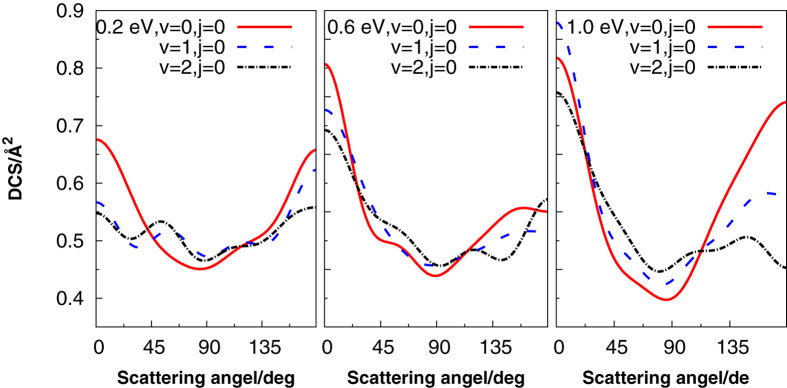
Differential cross section at different vibrational energy levels with fixed collision energies (0.2 eV, 0.6 eV, 1.0 eV).

The DCSs offer an excellent opportunity to study the most familiar vector correlation between the reagent and product relative velocity (*k* − *k*_0_). As shown in Fig. 7, it is clear that the DCS is dominated by scattering in both the forward and backward directions, which is consistent with the complex-forming mechanism for this reaction, and is similar to other exothermic reactions^39^,^40^. None of the DCS is completely symmetrical, showing slightly backward bias at all six collision energies. The reason for the results may be that with the energy increasing, and the trap binding becoming small, when the centrifugal potential is approximately equal to the depth of the well, the trend of head to head collision dominant the reaction. So, backward scattering is slightly larger than the forward scattering. The DCSs of different vibrational energy levels with fixed collision energies (0.2 eV, 0.6 eV, 1.0 eV) are also plotted in Fig. 8. We can see that the vibrational excitation DCSs in both forward and backward are lower than that of the ground vibrational state. So we can conclude that the SH vibration inhibits the forward and backward scattering in the same collision energy.

## Discussion

We have reported a globally accurate PES for the electronic ground state of HCS based on a wealth of *ab initio* energies calculated at MRCI(Q)/AVQZ and MRCI(Q)/AV5Z level of theory, which is expected to be realistic over the entire configuration space. Such raw energies are subsequently extrapolated to CBS limit. The properties of the major stationary points, including geometries, energies and vibrational frequencies have been characterized on the current HCS(*X*^2^*A*′) PES, showing good agreement with other theoretical and experimental results. The various topographical features of the current PES give an accurate description over the short and long range interactions. The present PES has been subsequently employed to carry out the QCT calculation of the ICS, DCS and the thermal rate constant for the reaction C(^3^*P*) + SH(*X*^2^Π) → H(^2^*S*) + CS(*X*^1^∑^+^). The present HCS(*X*^2^*A*′) PES is recommended for dynamic studies of C(^3^*P*) + SH(*X*^2^Π) reaction in more detail,

## Methods

### Analytical potential energy surface function

The APES for the 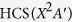 can be represented by MBE^41^, which is as follows:


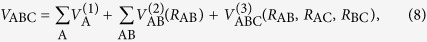


in which the one-body term 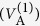 is the energy of the separated atoms in their corresponding electronic state, usually 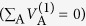 for all the atoms in their ground states, the two-body terms 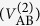 correspond to the diatomics PECs, including the nuclear repulsions, and the three-body term 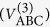 takes into account the interactions between three atoms.

For the HCS(*X*^2^*A*′) PES, it follows the dissociation scheme as


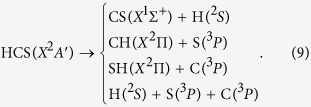


Since H(^2^*S*), C(^3^*P*) and S(^3^*P*) are all in their ground states, the values of 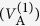 can be set to zero, which is similar to other systems^42^,^43^,^44^.

The terms 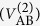 involve the 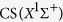, CH(*X*^2^Π) and SH(*X*^2^Π), with their analytical expression being represented by the formalism developed by Aguado and Paniagua^45^,^46^, and can be expressed as a sum of two terms corresponding to the short- and long-range potentials,


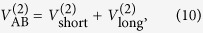


where


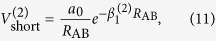


which warrants the diatomic potentials tending to infinite value when *R*_AB_ → 0, and the long-range potentials which tend to zero as *R*_AB_ → ∞ takes the following expression


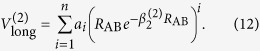


The linear parameters 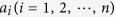 and the nonlinear parameters *β*_*i*_(*i* = 1, 2) in Eq. (11) and (12) are obtained by fitting the *ab initio* energies of the diatoms.

The three-body term 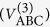 of the global potential in Eq. (8) can be written as *M*th-order polynomial^45^,^46^


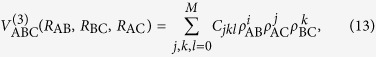


where 
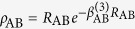
. 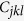 and 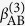 are linear and nonlinear parameters, which are determined in the fitting procedure. The constraints *j* + *k* + *l* ≠ *j* ≠ *k* ≠ *l* and *j* + *k* + *l* ≤ *M* are employed to warrant that the three-body term becomes zero at all dissociation limits and when at least one of the internuclear distances is zero. *M* is set to be 9 in the present work, which results in a complete set of parameters to be determined amounts to a total number of 192 for linear coefficients *C*_*jkl*_ and 3 for nonlinear parameters (i.e., 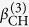, 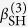 and 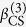). The root mean squared deviations (rmsd) values of the final PES with respect to all the fitted *ab initio* energies are gathered in Table 3. As shown in Table 3, a total of 5528 points have been used for the calibration procedure, thus covering a range up to 820 kcal/mol above the HCS global minimum. This table demenstrates that the final PES is able to fit the region of major chemical interest with a high accuracy (rmsd = 0.998 kcal/mol), including the global minimum and transition state for the C + SH dissociation process.

### *Ab initio* electronic structure calculations

The *ab initio* calculations have been carried out at the MRCI level^47^,^48^ of theory with the MOLPRO 2012 package^49^, including the Davidson correction [MRCI(Q)]^50^, using the full valence CASSCF^51^ wave function as the reference. The AV*Q*Z and AV5Z atomic basis sets of Dunning^52^,^53^ have been employed. A grid of 5528 *ab initio* points have been chosen to map the PES over the H − CS region defined by 2.0 ≤ *R*_CS_/*a*_0_ ≤ 4.5, 0.6 ≤ *r*_H−CS_/*a*_0_ ≤ 15, and 0.0 ≤ *γ*/deg ≤ 180, C − SH region by 2.0 ≤ *R*_SH_/*a*_0_ ≤ 4.0, 0.6 ≤ *r*_C−SH_/*a*_0_ ≤ 15, and 0.0 ≤ *γ*/deg ≤ 180, and S − CH region by 1.5 ≤ *R*_CH_/*a*_0_ ≤ 4.0, 0.6 ≤ *r*_S−CH_/*a*_0_ ≤ 15, and 0.0 ≤ *γ*/deg ≤ 180. *R*, *r* and *γ* are the atom-diatom Jacobi coordinates. *C*_*s*_ point group symmetry is employed in the *ab initio* calculation, which holds two irreducible representations, namely, A′ and A″. For 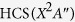, seven *A*′ and two A″ symmetry molecular orbitals (MOs) are determined as the active space, amounting to a total of 338 (207A′ + 131A″) configuration state functions.

### Extrapolation to CBS Limit

Subsequently, the *ab initio* energies calculated in this way were extrapolated to the CBS limit. The MRCI(Q) energy is treated as usual in split form by writing^54^


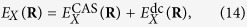


where the subscript *X* indicates that the *ab initio* energies are calculated in the AV*X*Z basis, and the superscript dc and CAS denote the dynamical correlation energy and complete-active space energy, respectively. Note that all extrapolations are carried pointwise, and hence the vector **R** of the nuclear geometrical coordinates will be omitted for simplicity. *X* = (*Q*, 5) is employed in the present work, which is denoted as USTE(*Q*, 5).

The CAS energies are extrapolated to the CBS limit by utilizing the two-point extrapolation scheme proposed by Karton and Martin^55^ and validated by Varandas^54^ in the extrapolation of the CASSCF energies


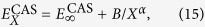


where *α* is a predefined constant. Being a two-parameter protocol (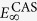, B), a minimum of two raw energies will be required for the extrapolation. Specifically, Eq. (15) will be calibrated from the CAS/AV(*Q*, 5)Z energy pairs, using a value of *α* = 5.34, which has been found to be an optimal value when extrapolating HF energies to the CBS limit.

The USTE method^54^,^56^ has been successfully applied to extrapolate the dc energies, with its formalism been written as


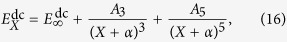


and here,


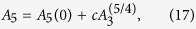


with *A*_5_(0) = 0.0037685459, *c* = −1.17847713 and *α* = −3/8 as the universal-type parameters^54^,^56^. Thus, Eq. (16) can be reduced into a (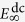, *A*_3_) two-parameter rule, which is actually used for the practical procedure of the extrapolation.

### Reaction Dynamics

By employing the present HCS(*X*^2^*A*′) PES, the reaction of C(^3^*P*) + SH(*X*^2^Π) → H(^2^*S*) + CS(*X*^1^∑^+^) is investigated by QCT^57^,^58^,^59^,^60^,^61^ calculations.

In present work, the collision energy is chosen to be 0.05–1.2 eV and batches of 100000 trajectories are run for each collision energy. The trajectories are initiated at a distance of 20 Å between the C atom and the SH diatom, and the integration step size is 0.1 fs. The maximal impact parameters *b*_max_ are optimized before running the trajectories. The ICS is then calculated as


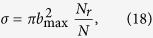


where *N*_*r*_ is the number of reactive trajectories in a total number of *N*, and *b*_max_ is the maximal value of the impact parameters.

The rate constant *k(T*) calculated with the ICS and Boltzmann integration over the collision energy is given by


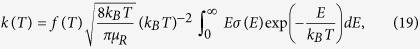


where *k*_*B*_ is the Boltzmann constant, *E* is the collision energy, and *f(T*) is the electronic degeneracies of reagents and products which can be expressed as ref. 16


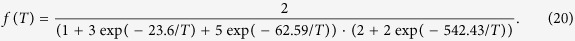


The distribution function *P(θ*_*r*_), which describes the *k*-*k*′ correlation, is expanded in a series of Legendre polynomials^57^,^62^,^63^


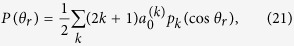


where


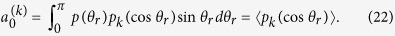


The coefficients 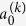 are called orientation parameter (*k* is odd) or alignment parameter (*k* is even).

## Additional Information

**How to cite this article**: Song, Y.-Z. *et al.* Globally accurate potential energy surface for the ground-state HCS(X^2^A′) and its use in reaction dynamics. *Sci. Rep.* **6**, 37734; doi: 10.1038/srep37734 (2016).

**Publisher's note:** Springer Nature remains neutral with regard to jurisdictional claims in published maps and institutional affiliations.

## Figures and Tables

**Table 1:** Spectroscopic constants of SH, CH, and CS diatoms, with the unit of *R*_*e*_ in *a*_0_., *D*_*e*_ in *E*_h_, and *ω*_*e*_, *ω*_*e*_*x*_*e*_, *α*_*e*_ and *β*_*e*_ in cm^−1^.

	*R*_*e*_	*D*_*e*_	*ω*_*e*_	*ω*_*e*_*x*_*e*_	*α*_*e*_	*β*_*e*_
SH(*X*^2^Π)						
MRCI(Q)/AVQZ	2.5388	−0.13827	2689.715	59.599	0.3051	9.5595
MRCI(Q)/AV5Z	2.5355	−0.13928	2695.140	59.405	0.3046	9.5844
MBE/CBS	2.5337	−0.14001	2698.763	59.254	0.3041	9.5982
Exp.^33^	2.5339	−0.13920	2695.8	59.9	0.270	9.4611
Theor.^22^	2.5354	−0.13987	2697.5	—	—	—
Theor.^28^	2.5325	−0.13953	2701.2	—	—	—
CH(*X*^2^Π)						
MRCI(Q)/AVQZ	2.1182	−0.13286	2842.397	69.265	0.5235	14.4307
MRCI(Q)/AV5Z	2.1171	−0.13332	2845.359	69.172	0.5229	14.4458
MBE/CBS	2.1162	−0.13374	2848.309	69.101	0.5223	14.4580
Exp.^33^	2.1170	−0.13377	2858.5	63.02	0.534	14.457
Theor.^29^	2.1170	−0.13326	2856.312	64.9321	0.5452	14.457
Theor.^30^	2.1180	−0.13285	2851.0	62.15	0.524	14.85
Theor.^31^	2.1172	−0.13396	2860	66	0.534	14.449
CS(*X*^2^∑^+^)						
MRCI(Q)/AVQZ	2.9181	−0.27276	1270.556	6.742	0.005842	0.8102
MRCI(Q)/AV5Z	2.9114	−0.27351	1277.433	6.796	0.005880	0.8140
MBE/CBS	2.9078	−0.27432	1281.150	6.816	0.005893	0.8160
Exp.^33^	2.9016	−0.2732	1285.08	6.46	0.005922	0.8200
Theor.^32^	2.9117	−0.2699	1278.00	6.4924	0.005837	0.8144

**Table 2:** Properties of stationary points on the fitted HCS(*X* ^2^ *A*′) PES (harmonic frequencies in cm^−1^).

Feature	*R*_1_/a_0_	*R*_2_/a_0_	*R*_3_/a_0_	*E*/*E*_h_	*ω*_1_	*ω*_2_	*ω*_3_
HCS Minimum							
GM/CBS	4.596	2.953	2.062	−0.3614	3775.10	704.03	995.84
GM/AV*Q*Z	4.598	2.966	2.058	−0.3589	3024	815	1214
GM/AV5Z	4.593	2.958	2.057	−0.3599	3024	810	1207
Theor.^16^	4.64	2.98	2.07	—	3000.4	888.96	1143.52
Theor.^15^	4.583	2.953	2.060	—	—	—	—
Exp.^19^	4.589	2.953	2.039	—	—	—	—
LM1	2.545	3.078	4.486	−0.3011	2739.45	2961.07	481.82
LM2	7.481	2.911	6.029	−0.2750	3909.03	105.82	35.49
LM3	6.655	2.909	9.564	−0.2746	19.41	3932.13	18.49
LM4	7.029	9.170	2.142	−0.1359	21.28	207.20	907.16
HCS Transition state (TS)							
TS1	6.144	2.911	4.356	−0.2739	386.87	3881.57	150.44*i*
TS2	2.632	3.193	3.024	−0.2666	2863.37	1446.62	429.55*i*
TS3	3.868	2.921	5.418	−0.2686	612.68*i*	3703.78	267.56
TS4	2.554	8.561	6.007	−0.1408	83.26*i*	2599.65	38.73
TS5	4.929	2.917	2.011	−0.3486	629.74*i*	3884.24	1088.12
TS6	2.687	5.045	2.358	−0.1423	645.74*i*	1946.23	584.65
TS7	3.021	3.037	6.058	−0.2544	808.55*i*	2593.46	225.44
HCS Second-order saddle (SP)							
SP1	4.046	6.288	2.242	−0.1207	309.01*i*	845.76*i*	867.87
SP2	2.587	5.590	3.003	−0.1379	2473.22	485.10*i*	270.75*i*
SP3	6.609	2.927	3.682	−0.2633	409.72*i*	3751.14	353.53*i*
SP4	3.484	2.976	3.653	−0.2509	3358.95	1221.41*i*	4399.20*i*
SP5	3.128	3.028	6.146	−0.2544	731.97*i*	2597.81	215.75*i*

**Table 3:** Root-mean-square deviations (in kcal mol^−1^) of the PES.

Energy	*N*^a^	rmsd	*N* > _rmsd_^b^
10	15	0.310	5
20	36	0.284	12
30	70	0.298	21
40	110	0.392	29
50	187	0.719	37
60	765	0.615	148
70	936	0.761	207
80	1085	0.794	251
90	1412	0.832	337
100	1897	0.819	544
200	4540	0.937	1119
400	4963	0.955	1249
600	5423	0.974	1362
820	5528	0.988	1393

^a^Number of points in the indicated energy range.

^b^Number of points with an energy deviation large than the rmsd.
